# Changes in Muscle Mass and Composition by Exercise and Hypoxia as Assessed by DEXA in Mice

**DOI:** 10.3390/medicina56090446

**Published:** 2020-09-03

**Authors:** Benjamin D. McNair, Nicholas A. Marcello, Derek T. Smith, Emily E. Schmitt, Danielle R. Bruns

**Affiliations:** Division of Kinesiology & Health, University of Wyoming, Laramie, WY 82070, USA; bmcnair@uwyo.edu (B.D.M.); nmarcell@uwyo.edu (N.A.M.); smithdt@uwyo.edu (D.T.S.); eschmit4@uwyo.edu (E.E.S.)

**Keywords:** exercise, muscle, hypoxia, dual x-ray absorptiometry, mouse

## Abstract

*Background and Objective:* Skeletal muscle is critical for overall health and predicts quality of life in several chronic diseases, thus quantification of muscle mass and composition is necessary to understand how interventions promote changes in muscle quality. The purpose of this investigation was to quantify changes in muscle mass and composition in two distinct pre-clinical models of changes in muscle quality using a clinical dual X-ray absorptiometry (DEXA), validated for use in mice. *Materials and Methods:* Adult C57Bl6 male mice were given running wheels (RUN; muscle hypertrophy) or placed in hypobaric hypoxia (HH; muscle atrophy) for four weeks. Animals received weekly DEXA and terminal collection of muscle hind limb complex (HLC) and quadriceps weights and signaling for molecular regulators of muscle mass and composition. *Results*: HH decreased total HLC muscle mass with no changes in muscle composition. RUN induced loss of fat mass in both the quadriceps and HLC. Molecular mediators of atrophy were upregulated in HH while stimulators of muscle growth were higher in RUN. These changes in muscle mass and composition were quantified by a clinical DEXA, which we described and validated for use in pre-clinical models. *Conclusions:* RUN improves muscle composition while HH promotes muscle atrophy, though changes in composition in hypoxia remain unclear. Use of the widely available clinical DEXA for use in mice enhances translational research capacity to understand the mechanisms by which atrophy and hypertrophy promote skeletal muscle and overall health.

## 1. Introduction

Skeletal muscle function is widely reported as critical for overall whole-body health [1]. Decreased muscle mass and function results in serious health concerns such as during age-related sarcopenia [2], cardiac [3] and cancer cachexia [4], and prolonged bed-rest [5]. In these clinical contexts, loss of muscle mass, strength, and quality is associated with functional decline, decreased mobility, increased fall and injury risk, and decreased quality of life [6,7,8]. Conversely, physical activity has been linked to improved inulin sensitivity [1], decreased cardiovascular disease risk [2], and overall bone health [3,4], suggesting exercise and physical activity as critical for attenuating chronic diseases, specifically musculoskeletal health. Understanding the mechanisms that contribute to gain and loss of muscle quality necessitates methods to accurately assess muscle mass/volume in both clinical and translational research settings. Despite the importance of monitoring muscle mass, few methods exist for murine models. Dual energy x-ray absorptiometry (DEXA) is the gold standard for the determination of body composition and is often used to measure changes in fat versus fat-free (muscle) mass [9] in clinical settings. To our knowledge, there are no previous reports validating this widely available clinical tool for use in pre-clinical models.

Among the known interventions to attenuate loss of muscle mass and/or stimulate muscle growth and function, regular exercise is among the most effective [10]. Exercise and physical activity have profound systemic and muscle-specific effects through stimulation of anabolic hormones and muscle protein synthesis [11], reduction of inflammation, improvements in muscle insulin sensitivity [12], and stimulation of mitochondrial biogenesis [13], all of which result in improved muscle size and function. Though several molecular mediators are activated in response to exercise, two widely acknowledged are the energy sensor 5′ AMP-activated protein kinase (AMPK) and peroxisome proliferator-activated receptor (PPARα) [14,15]. On the other hand, chronic hypoxia is implicated as both an independent cause of muscle loss as well as a contributing mechanism in several clinical disease states [16]. Hypoxia reduces muscle fiber size and muscle mass in mountaineers [17,18] as well as in rats chronically exposed to simulated hypobaric altitude [19], in part through induction of atrogene muscle atrophy F-box (Atrogin-1/MAFbx), and the negative regulator of muscle mass, myostatin [20].

We set out to assess changes in muscle mass and composition using two distinct interventions known to impact muscle and overall health: exercise and chronic hypoxia. We hypothesized that a clinical DEXA would permit accurate assessment of gain and loss of muscle mass with concomitant changes in muscle composition and expression of mechanistic mediators of these processes.

## 2. Materials and Methods

### 2.1. Mouse Models of Muscle Mass Loss and Gain

All experiments and methods described in this study were conducted in accordance with institutional guidelines and approved by the Institutional Animal Care Users Committee of University of Wyoming (protocol # A-3216-01). Adult (4–6 months) C57Bl6 male mice were randomly divided into one of three experimental groups. All experimental interventions persisted for four weeks. Control (CON; n = 6) mice were housed at Laramie altitude (~7200 ft; 2200 m). Loss of muscle mass was modeled by hypobaric hypoxia (HH; n = 9). HH mice were placed in a hypobaric hypoxia chamber (~17,000 ft; 5000 m; 10% O_2_) (Bruns Fabrication, Laramie, WY, USA). HH mice were removed from the hypoxic stimulus once per week for less than an hour for cage changes and data collection. Gain of muscle mass was modeled by voluntary free-wheel running (RUN; n = 6). Mice were provided free access to a running wheel. Daily distance run was recorded by number of daily revolutions and converted to kilometers (Columbus Instruments, Columbus, OH, USA). After four weeks, food was pulled the night before sacrifice and animals were humanely euthanized (FatalPlus; pentobarbital sodium). The lower hind limb complex (HLC) muscle, which consists of plantaris, gastrocnemius and soleus, was dissected from the rear limb. Quadriceps (Quad) muscle was dissected from the rear-limb. HLC and Quad muscles were weighed and flash-frozen for molecular analyses. Measurements of tibia length were performed by caliper to permit normalization of muscle weights to body size, not body mass.

### 2.2. DEXA Acquisition

DEXA were acquired on a GE Healthcare Lunar enCORE (Madison, WI, USA). Mice were anesthetized with isoflurane and placed stomach down with forelimbs taped perpendicular to the torso. Hind legs were gently pulled to straighten out the hips and adhered to the DEXA platform with the tail centered between the legs. In the DEXA program (GE Healthcare enCORE2011, Version 13.60, Madison, WI, USA), a new patient file was created for each mouse. Height and weight were set at the program minimum of 9.1 inches and 0.1 pounds, respectively. The mouse’s nose was placed slightly below the DEXA crosshairs, with the center line straight down the mouse’s back. Once the DEXA and mouse were in position, the scan parameters were set to 13.4 inches long and 9.1 inches wide, an area which only scans slightly larger than the entire animal. Under these parameters, the tip of the tail was not scanned. 

### 2.3. DEXA Analysis and Quantification

The first step in analysis was designation of anatomical regions. While for larger animals or humans the landmarks are very reliable, we opted for manual designation based on the accuracy of the program in identifying these landmarks in mice. The head region was determined by the lateral headlines and the neckline separator. The neckline was placed at the base of the head. The forearm line was angled to exclude the arm outside of the line. The area outside of these designations was labeled as “Arm” and was not included in analysis or calculations. Torso lines were adjusted along the sides of the body. Depending on the size of the mouse, the hip lines were manually widened to include the abdomen in the torso region. Spine lines were adjusted to include the spine. The hip line was placed at the top of the pelvis, dragging down the bottom to the base of the tail. Hip width was determined by stomach width to include the torso in the torso region. Leg regions were adjusted to include the whole back leg. Once regions were manually adjusted, tissue types were designated. The software automatically designates material as “bone”, “tissue”, or “air”. However, for mice, and likely other small rodents, the manual designation lacks specificity, thus we manually designated tissue types. The whole mouse was designated as soft tissue, based on the assumption that four weeks was not a long enough time to elicit changes in bone density [21,22]. The remainder of the scan area including the tail was designated as the material air. This manual editing and analysis is shown in Supplementary Figure S1.

### 2.4. Calculation of Changes in Tissue Fat and Fat-Free Mass

Once correct tissue designation has occurred, the program provides percent fat, lean muscle mass, fat mass, and overall tissue mass in all major regions based on previous anatomical designations. However, because the program uses initial body weight to derive tissue weight by regions, and the initial body weight is incorrect, as explained above, the tissue weights are also incorrect. Therefore, we used the % fat given by the DEXA discerning fat from lean muscle mass to calculate true changes in fat and fat-free losses within the leg and trunk.

### 2.5. Regulators of Muscle Growth and Atrophy

RNA was extracted from the HLC and Quad using standard Trizol protocols and reverse transcribed using an iScript cDNA synthesis kit (Bio-Rad, Hercules, CA, USA). Real-time RT-PCR was performed using iQ SYBR Green Supermix (Biorad) and normalized to the housekeeping gene 18S ribosomal RNA (18S). Data were expressed as ΔΔCt relative to Con. Primer sequences were as follows: 18S Forward: GCCGCTAGAGGTGAAATTCTTG, 18S Reverse: CTTTCGCTCTGGTCCGTCTT; atrogin-1 Forward: AGAAAAGCGGCAGCTTCGT, atrogin-1 Reverse: GCTGCGACGTCGTAGTTCAG; PRKAA2 Forward: GAGGCGGCCGAACAGG, PRKAA2 Reverse: GCACGTAGTGTCCGATCTTCAC; myostatin Forward: TCACGCTACCACGGAAACAA, myostatin Reverse: AGGAGTCTTGACGGGTCTGA; PPARα Forward: GACAGTGACAGACAACGGCA, PPARα Reverse: GTGGCAGGAAGGGAACAGAC.

### 2.6. Statistical Analysis

Body weight and weekly serial DEXA data were analyzed by a 1-way repeated measures analysis of variance (ANOVA). Terminal measurements were analyzed by one-way ANOVA with Fisher’s Least Significant Difference post-hoc analyses where appropriate. To further validate repeated measures assessments, delta body weight, trunk, and leg composition were calculated and compared to a reference value of zero by the one-sided Student’s t-test to determine if changes across the four week period were significantly impacted by RUN/HH (i.e., different from zero). Significance was set a priori at α < 0.05. Analyses were performed using SPSS Statistics Version 22 (IBM Corporation, Armonk, NY, USA). Data are presented as means ± standard error of the mean (SEM). 

## 3. Results

### 3.1. Changes in Body Weight with Free-Wheel Running and Hypoxia

**Figure 1:**
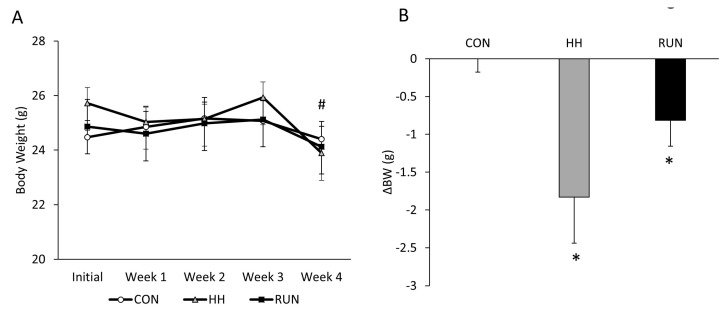
Changes in total body weight over four weeks in models of muscle gain and loss. (**A**) Body weight over four weeks was similar in Control (CON), hypobaric hypoxia (HH), and wheel running (RUN) mice. (**B**) Delta body weight (g) was significant in HH and RUN, with mice losing on average two and one gram, respectively. Serial changes in body weight progression were assessed by one-way repeated measures ANOVA and delta body weight by one-sided t-test compared to 0. Data are expressed as mean ± SEM. # *p* < 0.05 compared to Initial within RUN and HH. * *p* < 0.05 compared to 0. n = 6 CON, n = 6 RUN, n = 9 HH.

Changes in total body weight (BW) can influence gains and loss of muscle mass, thus we assessed changes in BW with free-wheel running and HH over the course of four weeks. Mice exposed to HH lost an average of two grams of BW while RUN lost an average of one gram of BW. CON mice remained weight-stable (Figure 1).

### 3.2. Temporal Changes in Trunk and Leg Fat by DEXA

**Figure 2:**
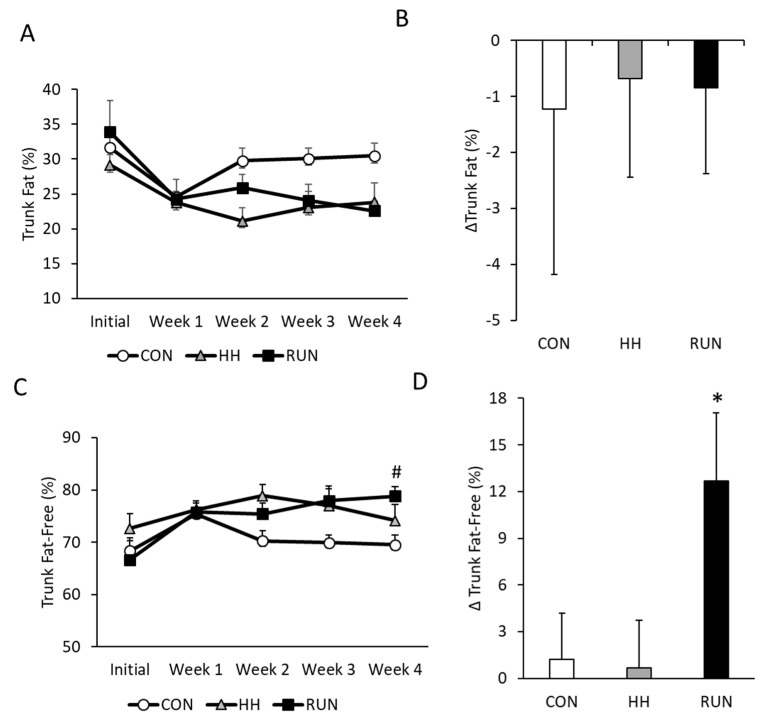
Changes in trunk composition over four weeks in models of muscle gain and loss. (**A**) Trunk fat was unchanged over four weeks and (**B**) no changes were found in delta trunk fat at study termination. (**C**) Trunk fat-free mass increased in RUN by week 4, but was unchanged in CON and HH, (**D**) with a significant gain in trunk fat-free mass at the end of four weeks in RUN. % Trunk fat and fat-free mass was determined by DEXA and changes were assessed by one-way repeated measures ANOVA with delta trunk composition assessed by one-sided t-test compared to 0. Data are expressed as mean ± SEM. # *p* < 0.05 compared to Initial within RUN. * *p* < 0.05 compared to 0. n = 6 CON, n = 6 RUN, n = 9 HH.

We assessed weekly changes in trunk and leg composition by serial DEXA analyses. At baseline, trunk composition was approximately 30% fat and 70% fat-free mass (Figure 2A,C) in all three groups. Trunk fat did not change in any of the experimental conditions (Figure 2A,B). However, by week 4, RUN gained trunk fat-free mass (Figure 2C) such that at the end of the experimental program, the RUN group had significantly higher fat-free composition (Figure 2D). 

**Figure 3:**
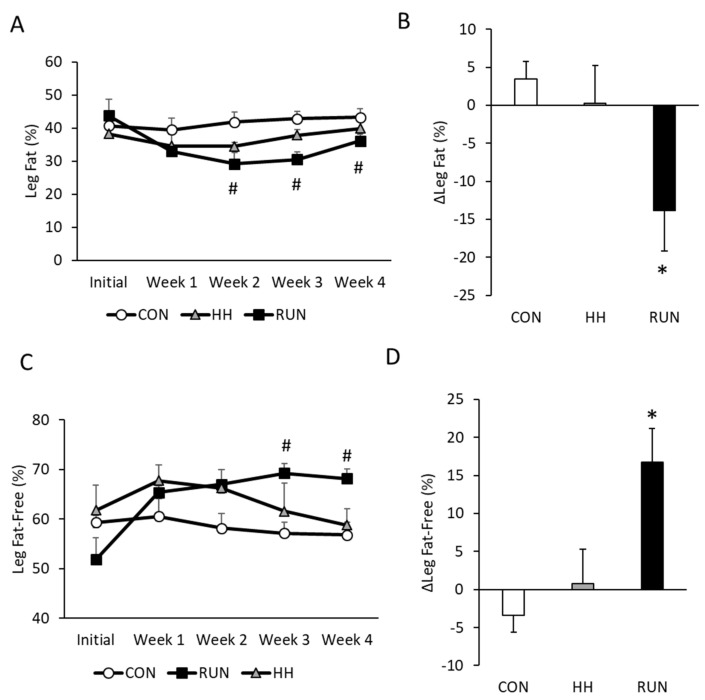
Changes in leg composition over four weeks in models of muscle gain and loss. (**A**) Leg fat decreased by three weeks in RUN with (**B**) delta (Δ) leg fat (%) lower at study termination in RUN. (**C**) Leg fat-free mass increased in RUN such that (**D**) by four weeks, Δ leg fat-free mass was significantly higher in RUN. % Leg fat and fat-free mass was determined by DEXA and changes were assessed by one-way repeated measures ANOVA with delta leg composition assessed by one-sided t-test compared to 0. Data are expressed as mean ± SEM. # *p* < 0.05 compared to Initial within RUN. * *p* < 0.05 compared to 0. n = 6 CON, n = 6 RUN, n = 9 HH.

In the leg, composition was approximately 40% fat and 60% fat-free mass (Figure 3A,C) in all three groups at baseline. Leg composition shifted with RUN, so that by the end of four weeks, fat-mass decreased and fat-free mass increased (Figure 3B,D). No changes in leg composition were noted in CON or HH conditions.

### 3.3. Hind Limb Complex and Quadriceps Mass, Fat-Free, and Fat-Mass

**Figure 4:**
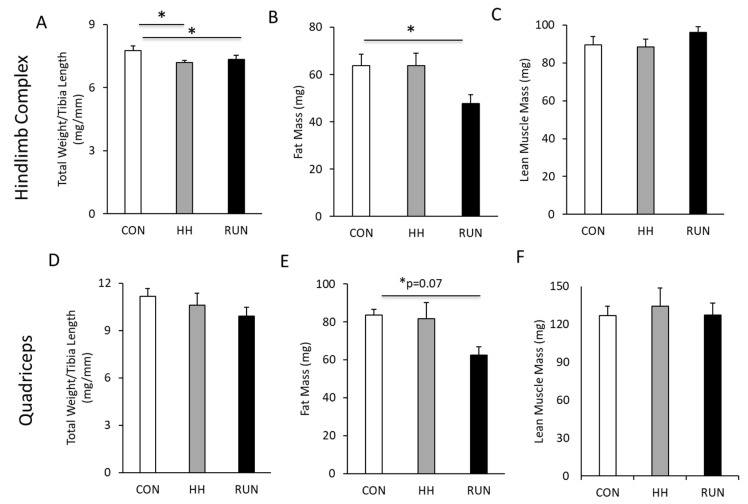
Terminal muscle masses, fat mass, and fat-free mass in models of muscle loss and gain. (**A**) Total hind-limb complex (HLC) weight was lower in HH and RUN compared to CON. (**B**) Four weeks of wheel-running decreased fat mass in the HLC, (**C**) while maintaining lean muscle mass. (**D**) Quadriceps total muscle mass was unchanged in HH and RUN compared to CON (**E**) but RUN elicited decreased quadriceps fat mass alongside (**F**) similar lean muscle mass. Data were assessed by one-way ANOVA. Data are expressed as mean ± SEM. * *p* < 0.05. n = 6 CON, n = 6 RUN, n = 9 HH.

At the end of four weeks, we dissected and carefully weighed the HLC and Quad. Total HLC and Quad mass were normalized to tibia length to account for animal size, not animal mass, given the significant changes in BW over four weeks. Total normalized HLC mass was significantly lower in HH and RUN compared to CON (Figure 4A). This loss of HLC mass in the RUN group was due to decreased fat mass (Figure 4B) with unchanged lean muscle mass (Figure 4C). Quad total mass normalized to tibia length was unchanged in HH or RUN (Figure 4D), with lower quad fat mass (Figure 4E) and no changes in lean quad mass (Figure 4F).

### 3.4. Molecular Mediators of Muscle Gains and Loss

**Figure 5:**
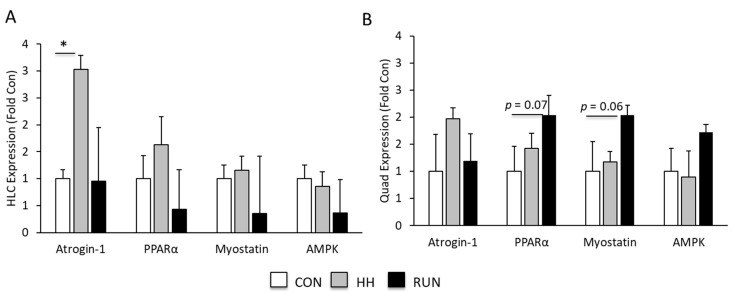
Molecular mediators of gain and loss of muscle mass. (**A**) HH stimulated expression of atrogin-1. (**B**) PPARα and myostatin expression were induced by RUN. Gene expression was quantified by qRT-PCR and normalized to 18S. Data are represented as fold from CON. Data were assessed by one-way ANOVA. Data are expressed as means ± SEM. * *p* < 0.05 compared to CON. n = 5 mice per group.

To assess the molecular mediators of muscle hypertrophy and atrophy, we quantified the expression of atrogin-1, myostatin, AMPK, and PPARα by qRT-PCR in the HLC and Quad. In the HLC, atrogin-1 expression was significantly upregulated in response to HH (Figure 5A), with no changes in expression observed in the other mediators of muscle mass. In the Quad, PPARα and myostatin tended to be higher in RUN than CON (Figure 5B).

## 4. Discussion

Skeletal muscle function is widely reported as critical for overall whole-body health. Understanding how physiological stimuli promote muscle health or muscle wasting (i.e., exercise and hypoxia, respectively) necessitates accurate measurement of changes in muscle mass and composition. Here, we set out to measure changes in muscle mass and composition in pre-clinical models as assessed by DEXA designed for clinical use. We found that free-wheel running induces muscle hypertrophy and loss of body fat and that hypobaric hypoxia induces muscle atrophy and loss of body fat. Furthermore, we report that these changes in trunk and leg mass and composition can be quantified by DEXA over the course of four weeks (summarized in Table 1). 

DEXA remains the gold standard for assessing body composition in humans [9], however, affordable pre-clinical versions are readily available and the widely available clinical tools have not yet been validated in pre-clinical models. Pre-clinical models provide a mechanistic understanding of disease and its attenuation by pharmaceutical and lifestyle interventions otherwise not possible in humans, thus quantifying changes in muscle mass and composition in mice is critical. Here, we report that a clinical DEXA accurately quantified changes in leg and trunk composition in response to free-wheel running and hypoxia. To validate this clinical tool in mice, we correlated dependent variables in the trunk and leg muscle (Supplementary Table S1). As anticipated, several variables were strongly associated such as average running distance and loss of trunk fat. Changes in leg fat % correlated with leg fat mass at sacrifice in both muscles, with stronger associations in the HLC compared to Quad. Therefore, changes in leg composition in these models appear to be more reflective of changes in the HLC, and not the Quad. This correlation is supported by our data, which demonstrated larger changes in the HLC than the Quad, and is interesting given the larger mass of the Quad than the HLC. While beyond the scope of the current investigation, this may suggest that the HLC muscles are either more subject to changes in composition or mass by these interventions, have a larger role in mouse ambulation/health, or both. These differences between muscle groups and the impact of exercise and hypoxia on their remodeling warrant future investigation. 

Exercise is a well-established positive regulator of muscle mass and function [23]. Therefore, we expected, based on considerable publications in healthy adult mice, to observe gains in leg fat-free muscle mass. Indeed, we observed increased total leg fat-free mass and decreased muscle fat mass. In the quadriceps, these changes were associated with higher expression of PPARα. Interestingly, running distance correlated with loss of Quad fat mass, but did not correlate with changes in HLC mass or composition, suggesting that running may elicit more profound changes in the Quad than the HLC. To our knowledge, no reports exist in pre-clinical models to understand how the Quad and HLC may differ with respect to muscle composition, but the precise mechanistic mediators behind these changes in response to voluntary exercise are of interest to our group. It is also likely that different modalities of exercise (i.e., resistance training or high intensity training) or different types of endurance exercise (i.e., forced treadmill training) would elicit different outcomes related to muscle mass and composition. 

Hypoxia, a known stimulator of muscle wasting, produced anticipated loss of HLC mass alongside 3-fold induction of atrogene expression with minor changes in Quad mass. We were unable to discern whether this loss of overall HLC mass came from fat or fat-free, as these were both unchanged over the course of four weeks. It is possible that HH stimulates overall loss of mass, rather than a shift in composition, as supported by previous reports in chronic hypoxia [24] and our mice, demonstrating significant loss of total body weight. Future work to understand the impact of hypoxia on muscle composition in diseases associated with systemic hypoxia (i.e., chronic obstructive pulmonary disease, heart failure) are of interest to our group and are of clinical significance. Of further interest is understanding the mechanisms by which hypoxia promotes muscle loss is critical for the design of strategies such as cardiovascular exercise or weight training to attenuate loss of muscle and overall health in these clinical conditions. Furthermore, understanding whether exercise can attenuate losses in muscle mass and/or changes in composition is also critical for the use of exercise as medicine in these conditions characterized by systemic or local muscle hypoxia.

We acknowledge several limitations in this investigation. In addition to our relatively small sample size, several other factors contributed to our conclusions. First, we only utilized male mice in the current study, however, female mice are known to respond differently to both wheel-running [25] and hypoxia [26] compared to males, thus the biological and physiological differences between males and females warrants future investigation. This validation study would also be strengthened by the addition of a gain of fat mass model such as obesity. Our mice ran considerably less than published average values in C57Bl6 male mice [27], likely due to several factors. First, our vivarium was located 7220 feet above sea level, a considerable hypoxic stress. To our knowledge, no reports have established normal running wheel distances at this elevation, though our group is currently establishing normative data as well as how running may change across generations of breeding at high altitude. Second, isoflurane was used to anesthetize animals for weekly DEXA. As seen in Supplementary Figure S2, this anesthetic reduces voluntary wheel-running distances for one to two nights after exposure. With repeated doses of isoflurane, the animals appear to demonstrate decrements in running performance and do not return to baseline levels. To our knowledge, the impact of isoflurane on running distances has not been previously reported, thus we encourage future investigations to take into consideration the impact of acute anesthetic exposure on voluntary physical activity. 

## 5. Conclusions

In conclusion, voluntary wheel running in mice promotes beneficial changes in skeletal muscle mass and composition, while hypobaric hypoxia induces profound loss of body weight and loss of muscle mass via unclear changes in composition. Our study validated a commonly used clinical tool (DEXA) for murine model use, suggesting that the clinical DEXA reliably detects skeletal muscle composition changes. This widely available tool may therefore be used in pre-clinical models of disease to understand the musculoskeletal system and its contribution to overall health. Future efforts to understand the mechanisms by which these changes in muscle mass occur are critical for the design of interventions to promote skeletal muscle and overall body health.

## Figures and Tables

**Table 1:** Summary of changes in body weight, muscle fat, and fat-free mass over four weeks of free-wheel running (RUN), hypobaric hypoxia (HH), or control (CON) conditions.

	BW	Trunk	Leg	HLC Mass	HLC FFM	HLC FM	HLC Gene Expression	Quad Mass	Quad FFM	Quad FM	Quad Gene Expression
CON	↔	↔	↔	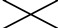	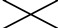	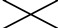	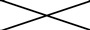	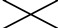	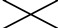	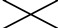	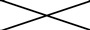
HH	↓ ↓	↔	↔	↓	↔	↔	↑↑ atrophy	↔	↔	↔	↔
RUN	↓	↑ fat-free	↑ fat-free	↓	↔	↓	↔	↔	↔	↓	↑ growth

CON mice demonstrated no change (↔) in BW or leg fat with an increase (↑) in trunk fat. HH mice saw no change in trunk or leg fat, but significantly lost (↓) body weight. RUN mice lost body weight alongside increased trunk and leg fat-free mass. HLC and Quad changes in composition and gene expression are normalized to CON, hence CON values are not reported. Four weeks of HH decreased HLC total mass without changing composition. In RUN, both quad and HLC fat-mass decreased. BW: body weight; HLC: hind limb complex; FFM: fat-free mass; FM: fat mass; Quad: quadriceps.

## Supplementary Materials

The following are available online at https://www.mdpi.com/1010-660X/56/9/446/s1, Figure S1: Editing of DEXA images, Figure S2: Running distances during the last two weeks of the 4-week experimental paradigm, Table S1: Correlation matrix between reported outcome variables.
