# Supplementary material for: Changes in Muscle Mass and Composition by Exercise and Hypoxia as Assessed by DEXA in Mice
Source: Medicina (Kaunas). 2020 Sep 3;56(9):446. doi: 10.3390/medicina56090446 (PMC7558449; doi:10.3390/medicina56090446)
Supplement: Supplementary file 1 [file medicina-56-00446-s001.pdf]

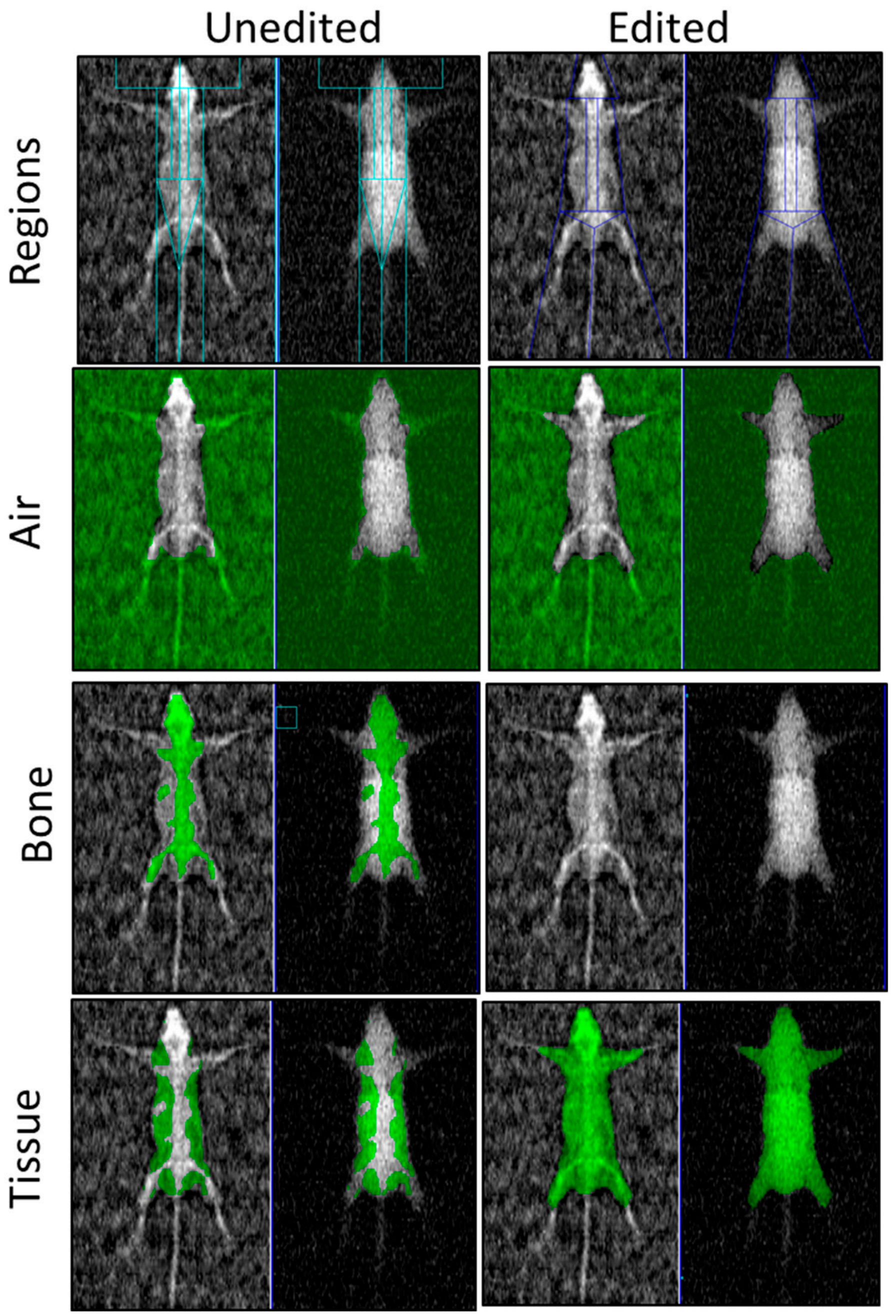

Supplemental Figure S1. Editing of DEXA images. Unedited DEXA scans are shown in the left two columns while edited DEXA scans are shown in the right two columns. The first column of both the Unedited and Edited groups shows bone while the second column shows tissue. The Unedited columns demonstrate what the software automatically designates as “regions” “air” “bone” and “tissue”. The Edited sections were manually moved to the correct positions to more accurately depict correct landmarks on the mouse anatomy. Validation of these measurements in the main manuscript supports the utility in manual editing, compared to the automatically generated designations from the program, originally designed for use in clinical models.

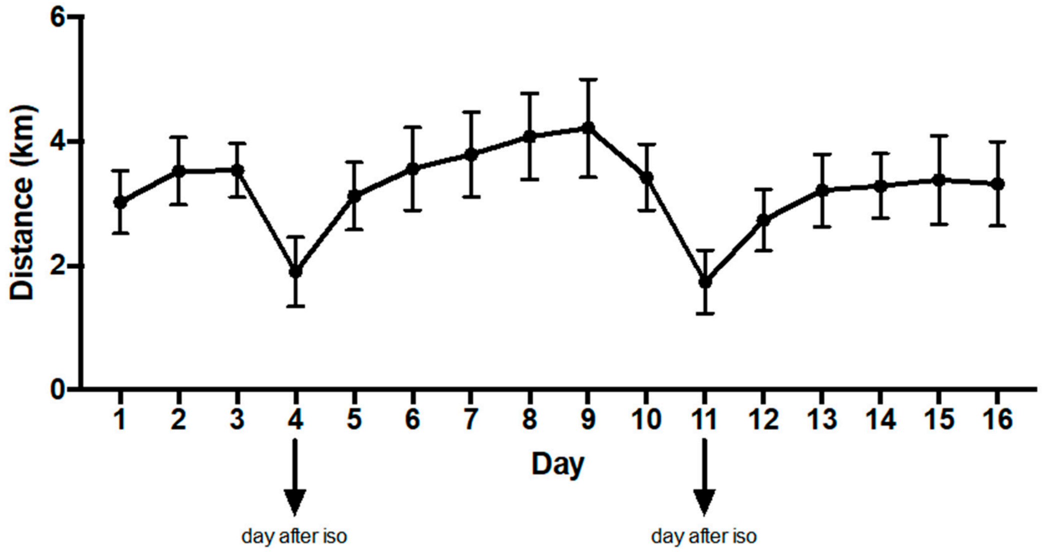

Supplemental Figure S2. Running distances during the last two weeks of the 4-week experimental paradigm. Mice were allowed to acclimate to the wheel for several days before data was recorded. During the final 16 days of wheel running, mice averaged 3.23 km/day ± 0.15 km. The dips in running wheel activity on days 4 and 11 are attributed to the day after the mice received isoflurane (ISO) as anesthesia for DEXA scans. n=5 runners.

	Avg Run (km)	Δ BW (g)	Δ Trunk Fat (%)	Δ Leg Fat (%)	Δ Leg Fat-Free (%)	HLC Mass (mg/mm)	HLC Fat Mass (mg)	HLC FFM (mg)	Quad Mass (mg/mm)	Quad Fat Mass (mg)	Quad FFM (mg)	HLC Atrogin	Quad PPARα	Quad Myostatin
Avg Run (km)	----	0.210	**-0.531**	-0.213	**0.605**	-0.2213135	-0.433	0.167	**0.551**	**-0.533**	-0.204	0.496	-0.345	-0.381
Δ BW (g)		----	0.195	-0.063	-0.245	0.329	0.052	-0.285	**0.542**	-0.064	**-0.670**	**0.637**	-0.084	0.117
Δ Trunk Fat (%)			----	0.102	-0.422	0.415	0.413	-0.203	-0.096	0.417	-0.269	-0.007	0.079	0.081
Δ Leg Fat (%)				----	**-0.589**	0.334	**0.818**	-0.578	-0.136	0.482	-0.113	0.090	0.254	0.392
Δ Leg Fat-Free (%)					----	-0.282	**-0.706**	**0.552**	0.347	**-0.782**	0.431	-0.021	-0.284	-0.401
HLC Weight (mg/mm)						----	**0.641**	0.221	0.000	0.180	-0.166	0.331	0.269	0.092
HLC Fat Mass (mg)							----	-0.472	-0.320	**0.651**	-0.111	0.160	0.460	0.548
HLC FFM (mg)								----	-0.011	**-0.677**	**0.688**	-0.060	-0.416	**-0.886**
Quad Weight (mg/mm)									----	-0.518	**-0.693**	0.412	-0.404	-0.262
Quad Fat Mass (mg)										----	-0.205	-0.157	0.332	0.504
Quad FFM (mg)											----	-0.415	0.038	-0.067
HLC Atrogin												----	0.027	-0.030
Quad PPARα													----	**0.697**
Quad Myostatin														----

Supplemental Table S1. Correlation matrix between reported outcome variables. Significant correlations ($p<0.05$) are noted by bolded text. n=5 CON, n=5 RUN, n=5 HH. Body Weight (BW), Hindlimb Complex (HLC), Quadriceps (Quad), Fat-free mass (FFM), Peroxisome proliferator-activated receptor (PPARα). Data were analyzed by Spearman's correlation coefficients.
